# Electron density is not spherical: the many applications of the transferable aspherical atom model

**DOI:** 10.1016/j.csbj.2022.10.018

**Published:** 2022-10-25

**Authors:** Marta Kulik, Paulina M. Dominiak

**Affiliations:** a Biological and Chemical Research Centre, Faculty of Chemistry, University of Warsaw, ul. Żwirki i Wigury 101, 02-089 Warsaw, Poland

**Keywords:** Transferable aspherical atom model, TAAM, Aspherical atomic scattering factors, Multipolar refinement, X-ray crystallography, Electron density, IAM, MATTS, Quantum crystallography

## Abstract

In the simplest approach and at low resolution, the electron density of an atom can be approximated by a sphere. However, the resolutions currently achieved in X-ray macromolecular crystallography reach atomic resolutions, where a more sophisticated approach is inevitable. This review summarizes the available electron density and scattering factor models and their applications with emphasis on the transferable aspherical atom model (TAAM).

## Highlights

• Electron density at high resolution should be modelled with aspherical approach.
• The transferable aspherical atom model (TAAM) is based on multipolar expansion.
• TAAM can rebuild the electron density and electrostatic potential of macromolecules.
• TAAM is also useful for the aspherical refinement and properties evaluation.

## Graphical abstract

**Figure:**
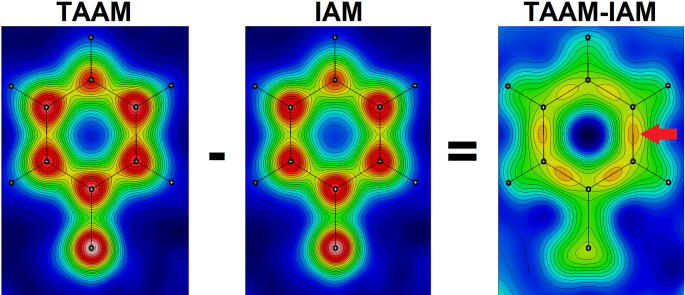

## 1 Introduction

Over the last more than 100 years, the world of X-ray crystallography has changed tremendously. The simplest model of electron density, called the independent atom model (IAM) [1], was proposed in 1915. Soon, the aspherical character of atomic density was noticed [2]. Technological advances have today made it possible to reach protein structure resolutions better than 0.5 Å. Key examples are crambin [3] and high-potential iron-sulfur protein [4]. At this resolution, the hypothesis that each atom can be modelled with a spherical electron density is no longer valid and should not be used. The departure from the spherical model of electron density becomes particularly obvious in regions with lone electron pairs but also chemical bonds and areas with secondary interactions, such as dipole–dipole interactions or hydrogen bonds. Nevertheless, in routine X-ray crystallography experiments IAM is still frequently used today to refine even relatively high-resolution models since it is much simpler to deploy than the more accurate approaches.

In this review we focus on the transferable aspherical atom model (TAAM), which is an alternative to using IAM for routine experiments, and can avoid the need to utilize more time-consuming methods that involve calculation of the wavefunction. Various applications of TAAM in modelling the electron density, refining the organic structures and analyzing the interactions between the components of biological systems are described. Special attention is given to the University at Buffalo Data Bank (UBDB), recently superseded by the Multipolar Atom Types from Theory and Statistical clustering (MATTS) data bank, which is currently the fastest developing data bank of the aspherical atom types in the field.

## 2 Approaches to model the electron density

**Fig. 1:**
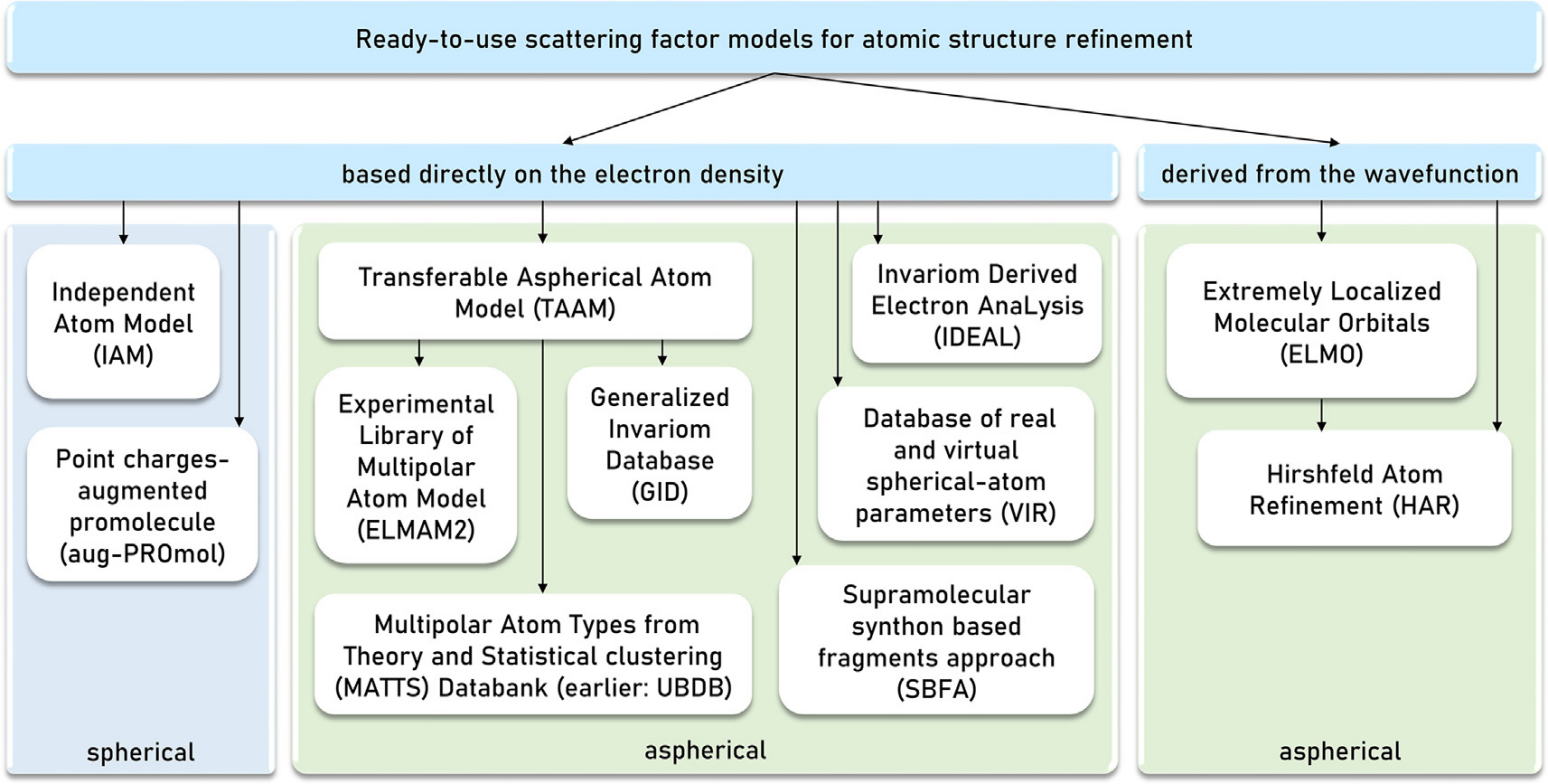
The main division of the scattering factor models containing all the parameters ready to use in the atomic model refinement.

When thinking about the electron density, one should start from the most basic concepts of the quantum mechanical approximations and methods, beautifully described for example in [5], [6]. The first one is the Born–Oppenheimer approximation, which allows us to separate the nuclear and electronic variables in the wavefunction. This approach simplifies the many-particle Schrödinger equation. Next, we can focus either on the molecular wavefunctions with their molecular spin orbitals in the Hartree–Fock (HF) method, or on the electron density as the central concept in density functional theory (DFT). One of the differences between those two concepts is that DFT considers the correlations between the electrons, while HF does not. In the post-HF methods, this HF missing feature is supplemented for example by application of Møller-Plesset perturbation theory or by Coupled Cluster and Configuration Interaction techniques. The accuracy of the chosen methods versus the computational cost is nicely sketched in [7]. The differences in the computational approaches impact the development of various libraries and methods depicted in Fig. 1.

From the point of view of a single atom, the scattering factor models based directly on electron density can be further divided as spherical and aspherical. IAM is among the spherical models. Another simple spherical charge-distribution model, being a result of the considerations on the charge penetration, is based on promolecules augmented with point charges named aug-PROmol [8]. Even though it models the electron density in a spherical way and it is not recommended for the atomic model refinement [9], it provides quite accurate estimates of the electrostatic energy in minimal computational time. A more accurate way to model the electron density is to use aspherical approaches. TAAM is an umbrella term for the data banks of aspherical atom types that can be transferred to chemically similar environments and share the same underlying methodology: Experimental Library of Multipolar Atom Model (ELMAM2) [10], [11], Multipolar Atom Types from Theory and Statistical clustering (MATTS) [12], [13], and Invariom database (GID) [14]. Even though the TAAM approach is more accurate than IAM and requires more parameters, the overall number of the parameters refined in both methods is the same since in the aspherical refinement the additional TAAM parameters are fixed and can simply be transferred between chemically similar systems. The existence of TAAM inspired other developments also based on the idea of transferability. The supramolecular synthon based fragments approach (SBFA) [15] exploits the fact that structural fragments representing supramolecular synthons can also be used to transfer the multipolar parameters. Another model features a combination of the real spherically-modeled atoms and additional scattering centers not located on the atoms – a database of real and virtual spherical-atom parameters (VIR) [16]. The database is dedicated to the construction of the electron density of proteins, nucleic acids and other small organic compounds. Finally, the recent development, called Invariom Derived Electron AnaLysis (IDEAL) [17], relies on Gaussian functions to reflect the deformation of electron density due to chemical bonding and lone electron pairs.

The methods based on the molecular wavefunction include Hirshfeld atom refinement (HAR) [18] and the libraries of Extremely Localized Molecular Orbitals (ELMO) [19]. Both approaches are intensively developed and extensively used for the reconstruction of the electron densities and for refinement of crystallographic models, including determination of the hydrogen atom positions [20], [21], [22], [23], [24], [25], [26], [27], [28].

The above-mentioned scattering factors are useful for the aspherical refinement of the atomic coordinates and displacement parameters using the standard quality X-ray crystallography data at atomic or near-atomic resolution. If the experimental X-ray diffraction data is of ultra-high quality and the resolution is better than 0.5 Å [29], then it is possible to refine not only the atomic model in the aspherical/TAAM refinement, but also the atomic and electronic model parameters in the multipolar refinement. Nevertheless, for multipolar refinement, refinement with TAAM can serve as a starting point [29]. Alternative methods to multipolar refinement are for example the X-ray constrained wavefunction fitting [30], [31] or using interatomic scatterers (IAS) in conjunction with IAM [32], [33]. Recent refinement frameworks include X-ray molecular orbital analysis [34] or a method based on semidefinite programming [35]. It is also possible to extract information about charge distribution directly from experimental data in a model-free manner using an information-theory-based technique called the Maximum Entropy Method [36].

## 3 The idea of TAAM

The valence electron densities are responsible for the major departures from the spherical character of electron density of an atom. IAM is a good approximation for the heavier elements due to the fact that the contribution of the core electrons to their overall density prevails over the contribution of the valence electrons. It is not true for the lighter elements. Moreover, in IAM, the atoms are assumed to be neutral or have an integer formal charge, expressed in electrons. To account for charge transfer between atoms, a small modification of IAM is necessary, known as the κ-formalism [37]:

(1) $$\rho (r)=P_{\mathrm{core}}\rho_{\mathrm{core}}(r)+P_{\mathrm{val}}\kappa^{3}\rho_{\mathrm{val}}(\kappa r)$$

In Eq. 1, $\rho_{\mathrm{core}}$ and $\rho_{\mathrm{val}}$ are normalized to one electron and multiplied by the electron population parameters $P_{\mathrm{core}}$ and $P_{\mathrm{val}}$. The parameter κ is introduced to account for the radial dependence of the valence shell. An additional aspherical multipole expansion term for valence electrons is introduced in the famous Hansen–Coppens equation [38]:

(2) $$\begin{array}{c} \rho (r)=P_{\mathrm{core}}\rho_{\mathrm{core}}(r)+P_{\mathrm{val}}\kappa^{3}\rho_{\mathrm{val}}(\kappa r)\\ +\overset{l_{\max}}{\underset{l=1}{\sum }}{\left(\kappa^{\prime }\xi \right)}^{3}R_{l}(\kappa^{\prime }\xi r)\overset{l}{\underset{m=0}{\sum }}P_{\mathrm{lmp}}d_{\mathrm{lmp}}(\theta ,\phi ) \end{array}$$

The first two terms are identical as in Eq. 1. Here, both the spherical valence and the aspherical valence electron density undergo expansion and contraction, which is described by the κ and $\kappa^{\prime }$ parameters, respectively. The population of multipole densities is denoted as $P_{\mathrm{lmp}}$, where *p* stands for the ± sign. The real spherical harmonics $d_{\mathrm{lmp}}(\theta ,\phi )$ is a density-normalized function. It is oriented in a local Cartesian coordinate system centered at the atomic nucleus. The $R_{l}(\kappa^{\prime }\xi r)$ Slater-type radial function dependent on the predefined values of ξ and $n_{l}$ is defined as:

(3) $$R_{l}(\kappa^{\prime }\xi r)=\frac{{\left(\kappa^{\prime }\xi r\right)}^{n_{l}}}{[n_{l}+2]!}\exp(-\kappa^{\prime }\xi r)$$

**Fig. 2:**
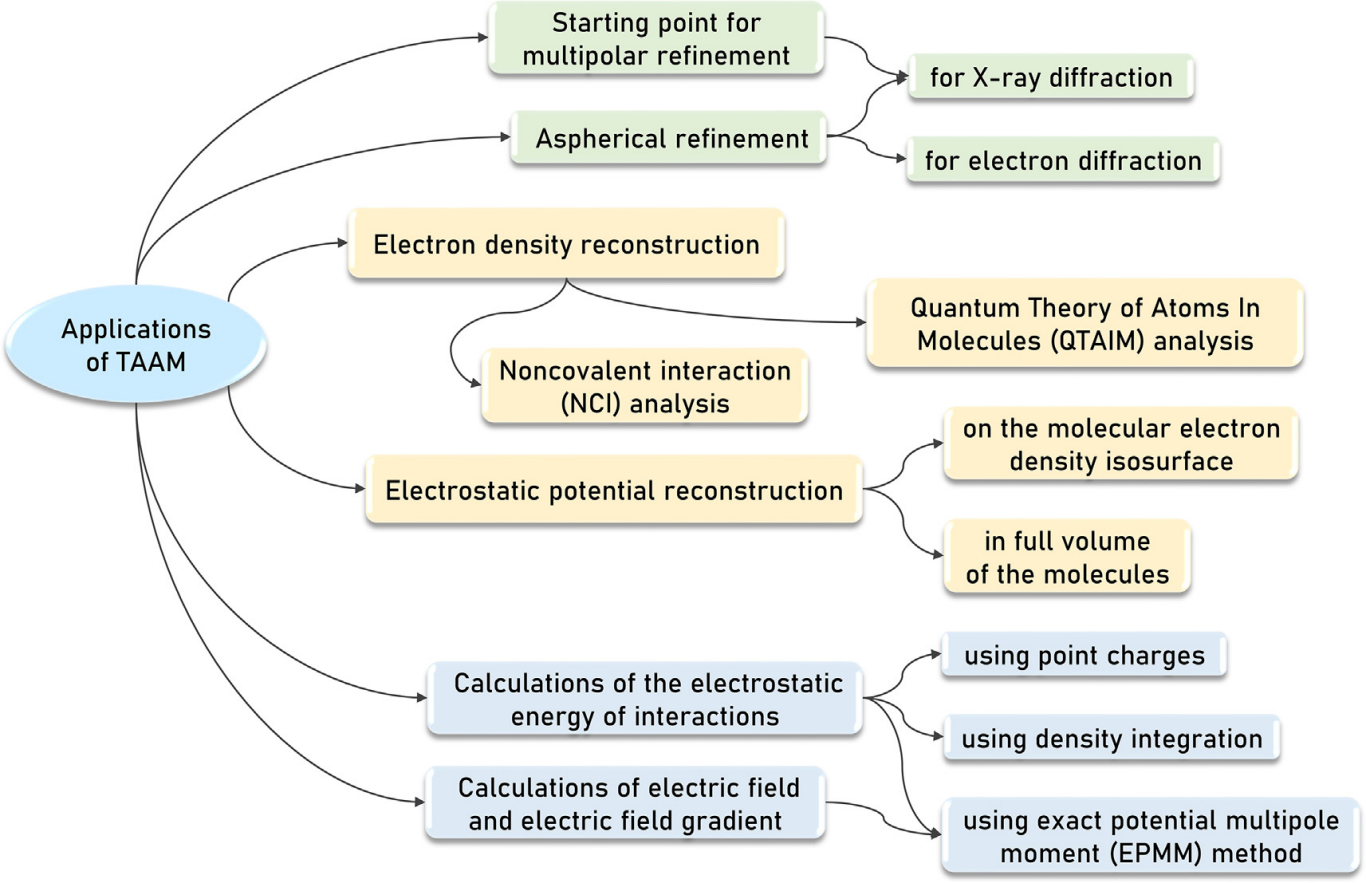
The main current applications of the transferable aspherical atom model (TAAM).

**Fig. 3:**
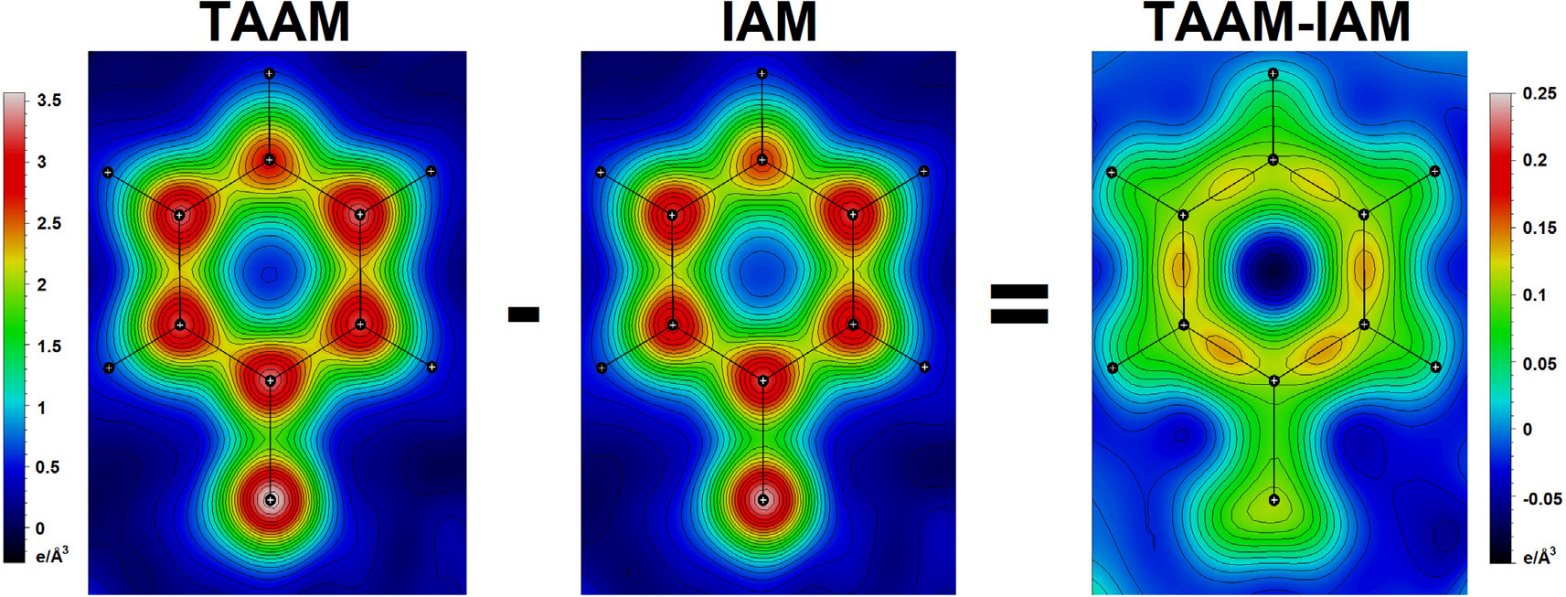
The 2D electron density maps at 1 Å resolution for phenylalanine from the lysozyme structure PDB entry 5k7o, calculated as described in [43]. The maps take thermal smearing effects into account. The scale for TAAM and IAM (left) is different from the scale for the deformation density map (right).

The idea of TAAM stems from the fact that the multipolar parameters derived for atom positions in one chemical environment, can be transferred to a different, similar chemical environment as they are effectively indistinguishable. It was first demonstrated for perylene, naphthalene and anthracene [39]. Later, it was found that the values of pseudoatom parameters from 2, calculated for corresponding peptide atoms, are typically almost identical [40]. Thus, they can be averaged and a data bank of experimental transferable density parameters can be created [40]. Currently, ELMAM2 [10], [11], MATTS [12], [13] and GID [14] are the three major data banks of the aspherical atoms types, based on the Hansen & Coppens formalism. Those databases differ in several ways, for example in the derivation of the structure factor or in taking into account the crystal-field influence [41]. All the data banks have found many applications, schematically shown in Fig. 2. A series of refinements using IAM and different TAAM approaches showed that the results were much better than the refinements done using IAM in terms of the quality of coordinates and thermal displacement parameters [41]. No significant differences were noted in the rates of reproducibility of geometries optimized in theoretical periodic calculations, such as the X-H bond lengths. It was also demonstrated that TAAM refinement with individual data banks significantly improves the discrepancy R factors [42]. The second important application of the data banks is rebuilding the electron density of the structures for which only the structural measurements were done and the charge-density data is not available. A comparison of the electron density description in TAAM (calculated with UBDB) and in IAM is shown in Fig. 3. Note the peaks visible on the covalent bonds in the deformation density map. Even though the aspherical models emerged in the X-ray crystallography field, they can also be used for electron diffraction and electron cryomicroscopy at high resolution.

Historically, the first database that emerged as a result of the considerations on the transferability of the atom types was ELMAM [40], [44], [45], which was restricted mostly to protein atoms. Later, it was expanded to include the atoms present in the most common organic molecules and improved to ELMAM2 [10], [11]. The definition of the local coordinate system was also changed to take into account the local symmetry. Both, ELMAM and ELMAM2, rely on the averaged multipolar parameters derived from high-resolution X-ray diffraction experiments but the algorithm of averaging the multipolar parameters over a family of atom types is different. The influence of the crystal field is in principle considered in the data bank. The lengths of the covalent bonds between non–hydrogen and hydrogen atoms during the multipolar refinement of the model molecules against the experimental data are constrained to the lengths observed in the neutron diffraction experiments. It stems from the fact that the electron density parameters cannot be refined together with the hydrogen atom positions since they are correlated. The data bank was mainly used for the TAAM refinement, where the multipolar parameters are copied from the data bank and are kept fixed during the refinement, while the atomic coordinates are being changed and there is no need to constrain the bonds involving hydrogen atoms. ELMAM/ELMAM2 can also serve as a starting point for the multipolar refinement of proteins with constraints [46]. The ELMAM/ELMAM2 data bank was used in many studies for the analysis of charge density and electrostatic interactions of organic molecules [47], [48], [49], [50], [51]. A large set of high-resolution protein structures was investigated using Bader’s quantum theory of atoms in molecules (QTAIM) and the noncovalent interaction (NCI) analysis [52].

The Invariom Database was also built on a similar idea as ELMAM and UBDB, except for the fact that the parameters of particular atom types are not a result of averaging but are instead obtained from single molecules [14]. Invarioms, or invariant atoms, are assigned to every chemically unique atom in a structure, taking into account the nearest neighbors and the bond order [53]. The multipolar parameters from Eq. 2 can be calculated not only for the experimentally-derived electron densities, but also for the electron densities calculated theoretically [54]. The atom types are capped with hydrogen atoms and their geometry is optimized. Invarioms have been used for example for molecular refinement [55], [56], [57], [58], [59], [60], to recreate and analyze the electron density [61], [62] and to investigate hydrogen atom positions in the intramolecular hydrogen bonds [63].

## 4 MATTS data bank

The University at Buffalo DataBank (UBDB) [64], [65], [66], [67] is currently developed under the name Multipolar Atom Types from Theory and Statistical clustering (MATTS) data bank [12], [13]. The MATTS data bank contains all atom types necessary to model proteins, nucleic acids and many other organic compounds, based on experimental geometries, extracted from the Cambridge Structural Database [68]. Those geometries are the starting point for the calculation of the molecular wavefunctions at the B3LYP/6-31G** level of theory. The static, valence-only structure factors are derived via Fourier transform of the molecular charge densities [69] and fitted with the Hansen-Coppens formalism. Theoretically derived phases of structure factor and, when necessary, local symmetry constraints are used in the refinement procedure. The monopoles, bond directed dipoles and quadrupoles of hydrogens are taken into account in the refinement, whereas the heavy atoms are refined up to the hexadecapolar level. Each entry to the database includes the information about the chemical environment of the atom, such as the number and element of the closest covalently attached neighbours, information about being a part of an aromatic ring etc. The intermolecular interactions are not taken into account. The covalent bond order is not explicitly specified, which makes the assignment of the atom types easier [13]. The parameters from the Hansen-Coppens’ equation are averaged over a family of chemically similar atoms. Both GID and MATTS parameters are derived from theoretical densities, which indicates the absence of any systematic/experimental errors, temperature or phase problems.

UBDB and MATTS have found a wide range of applications, such as in TAAM refinement [41], [70]. It has been demonstrated that using UBDB it is possible to locate the hydrogen atoms almost as precisely as using HAR [71]. A very rough estimation of the electrostatic energies of interaction for a biological system may be done using the classical Coulomb electrostatic interaction energy and simple point charges from any classical force field, such as Amber [72] or CHARMM [73]. On the other hand, for smaller systems, it is possible to perform advanced quantum mechanical calculations, which are much more accurate but extremely time-consuming when applied to the large biological systems. The multipolar approach allows the calculation of more accurate contributions to electrostatic energy of interactions than using simple point charges or IAM only in much shorter computational time than for quantum calculations, using TAAM in combination with the exact potential multipole moment (EPMM) method [74]. This method emerged from the evaluations of the accuracy of electrostatic energy derived from the electron densities from Eq. 2 using point multipole moment approximations compared to the quantum mechanics calculations for organic dimers [75]. Large discrepancies were observed for particularly short distances which led to application of the more time-consuming EP method for the contacts shorter than approximately 4.5 Å and the faster MM method for the long-range interactions [74]. It is worth mentioning that the UBDB/MATTS + EPMM interaction energy takes into account the unperturbed charge distributions of molecules in a complex [76], including charge penetration, and it corresponds to the first-order polarization energy in the Symmetry-Adapted Perturbation Theory (SAPT) of intermolecular interactions [77], [78], [79]. The exchange, induction and dispersion contributions to the intermolecular interaction energy are omitted. The UBDB/MATTS + EPMM method for the estimation of the electrostatic energy of interactions was applied to a range of systems, including proteins and nucleic acids [80], [81], [82], [83], [84], [85], [86], [87], [88]. The electron densities generated with UBDB were also used to calculate the electric field and electric field gradient in an infinite crystal for several small molecules [89].

Electrostatics often determines the strength and specificity of binding of ligands to their target receptors. A very simple analysis of the electrostatic potential of a molecule frequently involves showing only the values of the electrostatic potential on a molecular electron density isosurface, for example [11], [86], [61], [90]. Using UBDB/MATTS it is possible to correctly recreate the electrostatic potential not only at the surface of the molecule, but also within the full volume of the molecule by computing it by integration of the total electron density in direct space [67], [91], [92] or by Fourier summation in reciprocal space [43]. The latter study concerned the electrostatic potential density map calculations for two large protein systems.

## 5 Summary and outlook

This short review summarizes the electron density models used in X-ray crystallography and the applications of the aspherical transferable models. It is designed to be of practical use for readers dealing with X-ray or electron diffraction but not yet familiar with the choice of approaches more accurate than IAM for atomic model refinement and analysis.

With so many contributions in the field of electron density prediction with machine learning, it would seem that the existence of the data banks of the aspherical pseudoatoms is endangered. This is not the case since the aspherical pseudoatoms have several advantages over machine learning, widely discussed in [13]. Machine learning can be used in data bank creation, for controlling the number and the level of detail of the atom types, or checking for mistakes in the data used for the data bank preparation. On the other hand, machine learning in combination with chemical knowledge-based information can also be used to predict various features of the chemical compounds and their complexes.

## Funding

MK and PMD received funding from the National Centre of Science (Poland) through Grant OPUS UMO-2017/27/B/ST4/02721. MK acknowledges the support from the Ministry of Science and Higher Education through the programme “Excellence Initiative - Research University (2020–2026)” at the University of Warsaw.

## Declaration of Competing Interest

The authors declare that they have no known competing financial interests or personal relationships that could have appeared to influence the work reported in this paper.
